# The Preparation and Effects of Organic–Inorganic Antioxidative Biomaterials for Bone Repair

**DOI:** 10.3390/biomedicines12010070

**Published:** 2023-12-27

**Authors:** Qihao Guo, Shuoshuo Yang, Guoqi Ni, Jiale Ji, Mengwei Luo, Wei Du

**Affiliations:** 1 Key Laboratory of Textile Fiber and Products, Wuhan Textile University, Ministry of Education, Wuhan 430200, China; qhguo@wtu.edu.cn; 2 State Key Laboratory of New Textile Materials and Advanced Processing Technologies, Wuhan Textile University, Wuhan 430073, China; 3 Hubei Key Laboratory of Bioinorganic Chemistry & Materia Medica, School of Chemistry and Chemical Engineering, Huazhong University of Science & Technology, Wuhan 430074, China; niguoqi1999@163.com (G.N.); u201710480@hust.edu.cn (J.J.); weiyu6364917@163.com (M.L.); 4 School of Materials Science and Engineering, Wuhan Textile University, Wuhan 430200, China

**Keywords:** antioxidative materials, ROS, bone repair, osteogenesis

## Abstract

Reactive oxygen species (ROS) has great influence in many physiological or pathological processes in organisms. In the site of bone defects, the overproduced ROS significantly affects the dynamic balance process of bone regeneration. Many antioxidative organic and inorganic antioxidants showed good osteogenic ability, which has been widely used for bone repair. It is of great significance to summarize the antioxidative bone repair materials (ABRMs) to provide guidance for the future design and preparation of osteogenic materials with antioxidative function. Here, this review introduced the major research direction of ABRM at present in nanoscale, 2-dimensional coating, and 3-dimensional scaffolds. Moreover, the referring main active substances and antioxidative properties were classified, and the positive roles of antioxidative materials for bone repair have also been clearly summarized in signaling pathways, antioxidant enzymes, cellular responses and animal levels.

## 1. Introduction 

Reactive oxygen species (ROS) produced by cellular metabolism can act as signaling molecules to regulate many physiological [1] and pathophysiological [2,3] processes. However, the excessive generation of ROS induces oxidative stress (OS), resulting in cell damage, mitochondrial malfunction, and decreasing activity of intracellular antioxidant enzymes [4,5]. Moreover, the high level of ROS may cause mitochondrial DNA damage, chromosomal aberrations, and even apoptosis/necrosis [6]. 

The common ROS typically includes singlet oxygen (^1^O_2_), hydroxyl radicals (•OH), superoxide anion (O_2_^−•^), peroxyl radicals (ROO•), and hydrogen peroxide (H_2_O_2_) [7]. In the process of bone metabolism, normal cells could exhibit antioxidative function by generating many intracellular antioxidant enzymes [8] including catalase (CAT), glutathione peroxidase (GPX), superoxide dismutase (SOD), etc. In addition, the ways of cell-cell communication [9] and cell-extracellular matrix (ECM) [10] interaction might also reduce ROS formation during bone regeneration. Of note, OS showed great detrimental effects on osteogenesis by inhibiting cellular viability of osteoblasts, reducing activity of alkaline phosphatase (ALP) and decreasing the expression level of osteogenesis-related gene [11]. Furthermore, the overproduced ROS can further promote osteoclastogenesis and stimulate the activity of osteoclast to inhibit osteogenic differentiation, leading to osteoporosis with high bone loss level and low bone strength [12]. At the interface of bone tissue/implant, the large number of ROS produced in the bone defect [13] significantly reduces the osteogenic biofunction of implants, finally leading to the failure of osseointegration. For bone repair in diabetic environment, the SOD activity of organism was seriously limited with great ROS-caused oxidative damage, which was a special case of bone regeneration [2]. 

Many studies have demonstrated that antioxidants can not only prevent OS to maintain the activity of osteoblasts, but also promote osteogenic differentiation [14]. Antioxidants play a critical role in osteogenesis by regulating many signaling pathways that relate to ROS-induced oxidative injury [15,16]. For instance, resveratrol up-regulated the intracellular antioxidant enzymes (including CAT, SOD, and GPX), stimulating the SIRT1-mediated signaling pathway to protect osteogenic differentiation against oxidative damage [17]. In addition, antioxidants can promote the levels of expression of many osteogenic cytokines and osteogenesis markers [18]. For instance, ginsenoside-Rb2 successfully improved the expressions of mRNA of ALP, osteopontin (Opn), and osteocalcin (Ocn) against oxidative damage induced by H_2_O_2_ [11]. In fact, bone regeneration is a dynamic process of osteogenesis and osteoclasis. Interestingly, many antioxidants showed not only promoted osteogenesis, but also inhibited osteoclast differentiation in vivo and in vitro [19,20]. Moreover, antioxidants can also effectively reduce the negative effects of osteogenesis that is induced by wear debris particles of implants [21,22]. The antioxidative ingredients played an important role in bone repair, which shows important application prospects in the field of bone tissue engineering materials. 

With the development of research on bone injury, the preparation of osteogenic materials gradually pays more attention to the serious effects of OS microenvironment in bone defects in addition to focusing on traditional bone regeneration. Owning to the lack of antioxidant activity, many traditional bioactive materials including hydroxyapatite (HA) are not very suitable for the complex and serious environment of oxidative stress. It is necessary to prepare biomaterials with anti-OS activity for enhancing bone regeneration. Of note, antioxidative modification is an effective way to enhance the traditional bone repair materials. Currently, more and more researchers tend to choose osteogenic materials with antioxidative function to repair bone defects. So, the antioxidative bone repair materials (ABRM) emerged and rapidly developed. More and more attentions have been paid to the preparation and effects of antioxidative materials on osteogenesis. ABRM exhibits a very broad prospect of research and has made great progress in recent years. 

It Is significant to provide an insight into osteogenic materials with antioxidative function, which provides a guidance for design and preparation of bone repair materials in the future. Here, we introduced the recent types of ABRM including nano-scale materials, surface coatings and scaffolds that apply in bone repair. Moreover, we further classified and summarized the current active components for antioxidative bone repair, and their effects of antioxidant materials in enzyme activity, free radical scavenging, in vitro cell response and in vivo osteogenic mineralization. 

## 2. Types of Antioxidative Bone Repair Materials in Application

**Figure 1:**
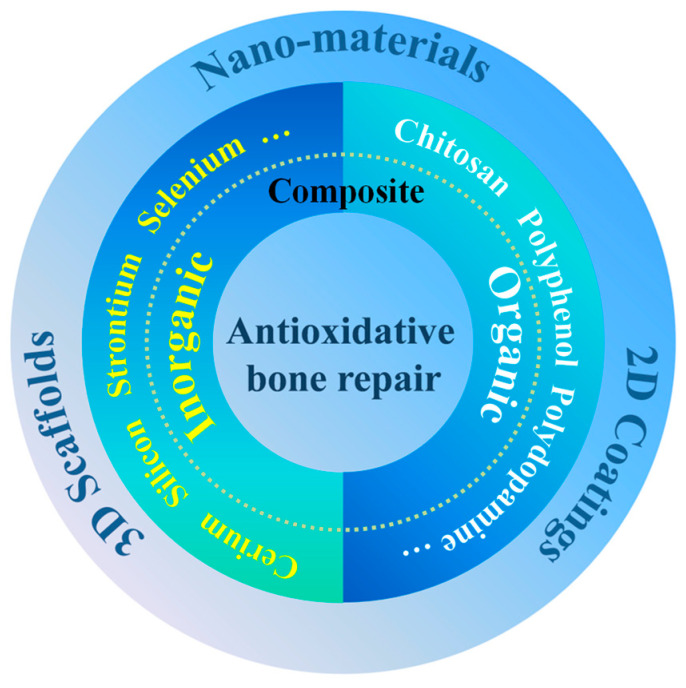
Classification of antioxidative materials for bone repair.

ABRM has been widely fabricated for bone repair in different scales (from micro to macro), including nanoscale materials, 2-dmensional (2D) coating and 3-dimensional (3D) scaffold, as shown in Figure 1. 

Nanometer materials with high specific surface area are usually within 1 to 100 nm (at least in one dimension), which possess many special chemical and physical properties [23]. Interestingly, natural bones are composed of various composite nanomaterials with multi-stage and self-assembly structures [24]. From the point of view of bionics, nano-biomaterials may exhibit some special biological activities in bone repair. As a main mineral component of bone tissues, calcium phosphates (CaP) is nanostructured and embedded in soft matrix of collagen. In nanomedicine, CaP-based biomaterials mainly include tricalcium phosphate (TCP), amorphous CaP, and HA [25]. HA (stoichiometric formula: Ca_10_(PO_4_)_6_(OH)_2_, Ca/P = 1.67) is a rod-like CaP with osteoconductivity, biocompatibility, osteoinductivity, and bioactivity. In native bones, the physical dimension of HA is about 100 nm in length, 20–30 nm in width and 3–6 nm in thicknesses [26]. Compared with unstable amorphous CaP and TCP, HA in crystalline phase was very stable in acidic environment [27]. In addition to the conventional hydrothermal method [28], the template methods were also widely used to manufacture HA crystals via biomineralization [29,30]. Of note, the manufacture strategy of composite templates may obtain more excellent HA crystals with precise nature structures [31,32]. As the mineral crystal of natural healthy bone, HA has been extensively used in bone repair [33,34]. However, many traditional osteogenic nanomaterials usually lack antioxidative activity, which limits their further applications. Currently, there is research on the antioxidative modification of osteogenic nanomaterials to endow traditional osteogenic materials with antioxidative function and further enhance their osteogenic activity. The general strategy is to use the traditional nanomaterials as carriers for doping modification, or to modify their surface by loading antioxidants. For instance, polyphenol is widely used as organic antioxidant, playing an important role in antioxidative modification of nanomaterials. Some research showed that the introduction of quercetin (a polyphenol drug) by doping or adsorption method can effectively enhance the antioxidative activity of HA [35,36]. In addition to HA, bioactive glass (BGs) is also an important traditional biomaterial for osteogenesis. Considering the complex environment of oxidative stress in bone defects, it is significant to construct antioxidative BGs-based nano-materials. In order to enhance the antioxidative function of BGs, Zheng et al. prepared Ce incorporated BGs nanoparticles via doping method [37]. Owning to the capability of fast valence switch, Ce-based inorganic antioxidants exhibited great antioxidation, which shows significant potential in combination with nanomaterials for bone repair. In fact, many noble-metal-based nanomaterials such as nanoparticles of Au and Ag can induce the production of ROS in cells to some extent, which limits their application in bone repair. Interestingly, the above negative effects of those noble-metal nanomaterials can be regulated by the method of antioxidative treatment [38,39].

**Figure 2:**
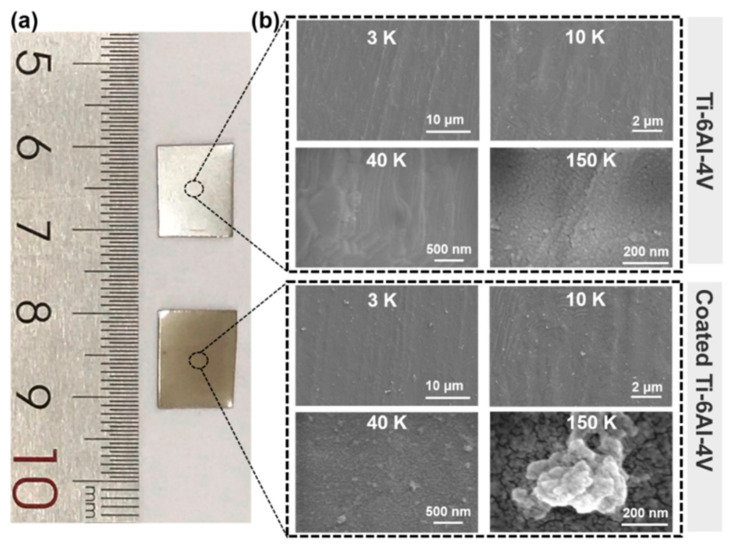
Physical maps (**a**) and SEM images (**b**) of titanium alloy (Ti-6Al-4V) implants before and after coating modification via Layer-by-Layer assembly of TA and drug loaded BSA [41], Copyright, 2023, Elsevier, License Number 5627500975577.

Metal materials with high mechanical properties were typically used as bone-replacement and bone repair materials, including stainless steel, titanium (Ti) alloy, cobalt alloy, etc. Owing to its good biocompatibility and bone-like mechanical properties, Ti implants have been widely used in bone tissue replacement. However, the surfaces of these conventional implanted materials generally lack sufficient osteogenic bioactivity. During the healing time of bone repair, Ti implants were usually mechanical chimerism rather than chemical osseointegration on the interface of Ti implants/bone tissue. It is necessary to modify the surface of implants to improve their osteogenic ability. In recent years, the antioxidative coating modification (including organic, inorganic, or organic–inorganic composite) on implant surfaces have been drawing much attention because of its promoting effect of osteogenesis. For instance, the organic composite coating of polyphenol-protein can reduce the negative effects of OS and promote the regeneration of bone tissue [40]. For the surface modification of Ti scaffolds, Yang et al. prepared a drug loaded coating of polyphenol-protein with good antioxidative and osteogenic bio-functions via both desolvation and Layer-by-Layer methods (Figure 2) [41]. The coating modification based on Layer-by-Layer technology is an effective and simple strategy to improve the antioxidative performances of Ti implant. Moreover, via magnetron sputtering or plasma spraying, the inorganic antioxidative coatings can be modified on the surface of Ti implants to enhance bone integration [42,43]. In addition, the strategy of organic–inorganic composite modification may also be an important way to construct antioxidative coatings for bone repair. As reported, the bioactive SiO_2_ coatings incorporated with antioxidative vitamin E were prepared via sol-gel method [44]. Vitamin E, an effective and useful exogenous antioxidant, in SiO_2_ coatings can induce the activity of SOD to decrease ROS production and accumulation [44]. The modified implants with bioactive antioxidative coatings can maintain excellent mechanical performances of original implants and combine the characteristics of modified bio-coatings.

Scaffold materials, as important frameworks for bone tissue regeneration, provide effective space for osteoblasts to adhere, grow and mineralize in complex processes of bone repair. Considering the positive effect of antioxidative property on osteogenesis in vitro and in vivo, it is of great significance to enhance the antioxidative bioactivity of scaffolds as a whole to promote bone repair in long-term. The antioxidative scaffolds can effectively regulate the surrounding microenvironment and regulate the response of osteoblasts. Polymer scaffolds usually have many advantages such as good biocompatibility and biodegradability, which were used as bone repair materials. Interestingly, a variety of antioxidants can be efficiently loaded in polymer scaffolds during the process of preparation. At present, there are two common methods to achieve the antioxidative scaffolds: (i) direct antioxidative modification of scaffold frameworks; (ii) and introduction of antioxidative bioactive components into scaffolds that can be released controllably. On the other hand, owing to high elasticity and strong plasticity of organic polymer materials, antioxidative scaffolds for bone repair can be designed in specific macro-shape (such as reticular and columnar) by mold or 3D printing technology [45]. Of note, inorganic scaffolds exhibit strong support and high strength that is close to the hardness of bone tissue. Moreover, the common inorganic bone repair scaffolds are BGs and bioactive ceramics. The BGs are proven to have excellent property of biomineralization [46], and exhibit good angiogenic potential base on the release of calcium ions and silicate [47]. However, those traditional inorganic BGs scaffolds always lack antioxidative activity, which were difficult to deal with the complex OS environment. Currently, the antioxidative incorporation, a simple and effective method, was mainly used to achieve the antioxidative function of inorganic scaffolds. For bone repair scaffold, Kaur et al. prepared CeO_2_ incorporated bioactive ceramics (SiO_2_-P_2_O_5_-MgO-CeO_2_-CaO) by sol-gel synthesis technique [48]. The Ce incorporated bioactive ceramic combined the supporting role of inorganic bone repair scaffold and Ce-based antioxidant activity [48]. To promote antioxidative activity and bone regeneration, Boulila et al. prepared silica-based BGs scaffolds introduced with polyvinyl alcohol (PVA) and ciprofloxacin (Cip) [49]. Owing to combing both advantages of inorganic and organic materials, the inorganic and organic composited antioxidative scaffolds may have great potential in the future.

## 3. Antioxidative Components for Bone Repair

Evidence suggests that antioxidations can regulate the process of bone repair, and ABRM have received increasing attention. ABRM are usually composite materials with antioxidants and osteogenesis functions. As shown in Figure 1, the antioxidative components mainly include (1) organic antioxidants such as polyphenol, dopamine, chitosan (Cs), etc.; (2) inorganic antioxidants such as cerium (Ce), strontium (Sr), selenium (Se), silicon (Si), etc.; and (3) organic and inorganic composite antioxidants. Moreover, owning to great antioxidative activity, vitamin C, vitamin E, glutathione, N-acetylcysteine, etc. were also prospective types of molecules to prepare ABRM.

### 3.1. Organic Antioxidants

(1) Polyphenol-based antioxidants

**Figure 3:**
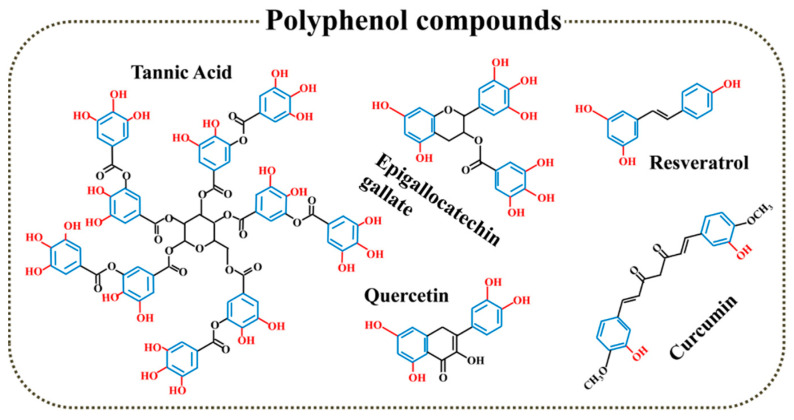
Polyphenol compounds with antioxidant function.

Polyphenol, as a famous natural antioxidant, is known for phenol groups in its chemical structure that can scavenge ROS by conversing phenol to quinonyl group under OS environment (Figure 3). Moreover, Polyphenol is widespread and biodegradable, which can be easily extracted from various natural plants. Currently, natural and synthetic polyphenols are widely used in bone repair including surface modification of nanomaterials, preparation of 2D coatings and 3D scaffolds for bone repair.

**Figure 4:**
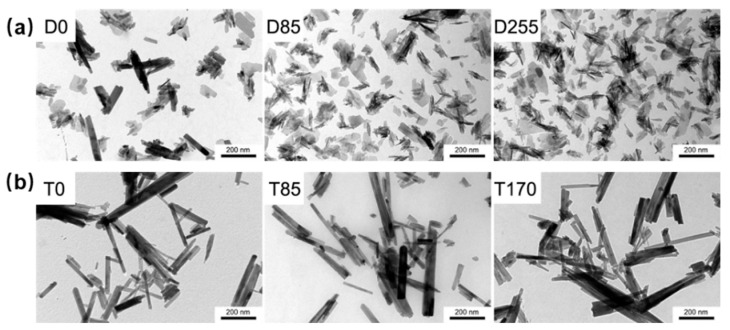
TEM images of the quercetin loaded HA nanocrystals products obtained by (**a**) direct synthesis (D0, D85 and D255) and (**b**) synthesis of monetite (T0, T85 and T170) [36], Copyright, 2016, Elsevier, License Number 5627520715422.

Polyphenol modification is a useful strategy to enhance the antioxidative biofunction of traditional bone repair materials. As shown in Figure 4, Forte et al. synthesized quercetin loaded HA nanocrystals for enhancing the anti-osteoporotic, antioxidative and anti-inflammatory properties of HA [35]. In order to improve the antioxidative activity of HA, Forte et al. further synthesized functionalized HA with antioxidative quercetin [36]. The doped quercetin did not significantly affect the structure of HA, and successfully maintained good antioxidative activity of free radical scavenger of polyphenol [36]. In bone tissue engineering, mesoporous hydroxyapatite (MHAP) is a special HA with mesoporous hierarchical architecture, which is similar to the structure of cancellous substance. Interestingly, this special porous structure with high surface area and porosity exhibited many advantages of promoting cellular attachment, proliferation, and differentiation [50]. In order to further enhance the antioxidative bioactivity of MHAP, Sistanipour et al. fabricated catechin conjugated MHAP (Cat@MHAP) based on amine-functionalization by a stable peptide bond [51]. Cat@MHAP is a novel efficient nano-antioxidant to enhance the repair of bone defects after surgery of osteosarcomas treatment according to its dual function of regulating cell proliferation of normal/tumor cells and increased osteogenic differentiation [51]. Based on physical doping, surface adsorption, and covalent grafting methods, the natural polyphenols were used to improve the antioxidative properties of HA-based bone repair materials. Moreover, the doping method may be more helpful to realize the large load of natural polyphenols.

Currently, more and more studies have shown that polyphenols-based coatings have important biofunction for bone repair. Moreover, the polyphenol-based antioxidative coatings can be prepared using simple and efficient methods [52]. In order to improve bone regeneration, Catechin hydrate, a representative natural plant flavonoid, was precisely self-assembled on the surfaces of many polymer scaffolds [53]. The polyphenols-based polymerized coating mainly relied on physical interactions (π-cation) rather than chemical polymerization, and showed special nano/microscale structures [53]. Through a simple and cheap dip method, Perikamana et al. prepared a bio-inspired (one-step) epigallocatechin gallate (EGCG) coating via cation (Na^+^)-induced coordination interactions and self-oxidative polymerization on various hydrophobic polymer substrates [54].

**Figure 5:**
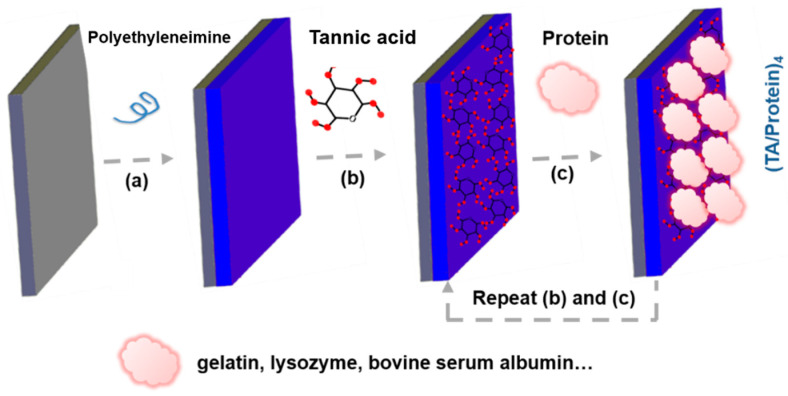
(**a**–**c**) Schematically illustrating fabrication process of (TA/Protein) coatings for antioxidative bone repair.

Tannic acid (TA), a famous natural polyphenol with great antioxidative activities [55], possesses numerous phenolic hydroxyl (Ph-OH) groups that can scavenge various ROS by conversion of Ph-OH to quinonyl group in OS environment. Of note, it is very convenient to construct protein-based antioxidative coatings for bone repair according to the interaction between TA and proteins. In previous studies, Yang et al. prepared many TA/protein coatings with good antioxidant property and biocompatibility via Layer-by-Layer method (Figure 5) [40,41,56]. Those TA/protein coatings can effectively promote cell attachment, osteogenesis and even bone regeneration. In addition, TA, as a cross-linking agent, can combine with other bioactive components to further improve the bio-function of protein-based antioxidative coatings. For instance, Zhu et al. fabricated nanohydroxyapatite (nHA) doped TA/Gel coatings to deal with OS environment around implants for bone formation [57]. Interestingly, TA can combine with antibiotics as well. An antioxidative polyelectrolyte coating was fabricated by alternately assembling TA and gentamicin sulfate (GS), an effective antibiotic, on the surface of electrospinning nanofiber of chitosan-polycaprolactone (PCL) [58]. So, polyphenols can be directly modified on the surfaces of implants or combine with a variety of bioactive substances (including proteins and antibiotics) to construct different forms of antioxidative coatings for bone repair. Interestingly, natural polyphenol with pharmacological activity can be embedded in scaffolds to construct an implantable delivery system as well. For ameliorating the water solubility and pharmacological activity of curcumin (Cur), an antioxidative polyphenolic pigment extracted from turmeric, Li et al. synthesized Cur-incorporated poly-lactic-co-glycolic acid (PLGA) microspheres via process of single emulsion solvent evaporation [59]. For bone repair in diabetic conditions, the stable Cur-loaded PLGA microspheres were further mixed into a composite scaffold of collagen/nano-hydroxyapatite (CHA) to regulate the process drug release [59].

In addition to natural polyphenols, many synthetic polyphenols were also applied to the preparation of ABRM. Chen et al. built a Cs derivative conjugated with catechol group (Chi-C) to improve the antioxidative efficiency of Cs-based coatings [60]. The catechol grafted Cs were further combined with gelatin (Gel) via Layer-by-Layer method to construct functional polyelectrolytes multilayer [60]. Moreover, the needle-like HA nanofibers was used as embedded agents to imitate the HA crystals in bone tissue [60]. Some chemically modified Cs porous scaffolds with polyphenol derivatives were synthesized via an acid-soluble/alkali-insoluble process to decrease intracellular level of ROS, which were beneficial for the enhancement of in vitro osteogenic differentiation of human adipose-derived stem cells (hADSCs) [61]. The antioxidative activity of modified Cs to scavenge 2-diphenyl-1-picrylhydrazyl free radical (DPPH•) radical was mainly resulted from the introduction of phenolic hydroxyl group and double bond [61]. In addition, the natural resveratrol (Res) possesses great activities of antioxidation, anti-inflammatory and immunmodulatory, and was grafted to polyacrylic acid (PAA). Moreover, the synthetic PAA-Res was further mixed into atelocollagen (Coll) hydrogels [62]. The composite (Coll/PAA-Res) scaffold showed great cytocompatibility, anti-inflammatory action, and excellent mechanical properties comparable to healthy cartilage tissue [62]. Additionally, the oxidative polymerized tyrosol in linear oligomeric mode (oligotyrosol and OligoTyr) was built via horseradish peroxidase/H_2_O_2_ reaction [63]. The high antioxidative capacity of OligoTyr partially resulted from the resonance stabilization of the aromatic rings in oligomeric structures [63]. Moreover, the antioxidative oligotyrosol was further incorporated into highly porous 3D polylactic acid (PLA) scaffold to regulate the release behavior of the active polyphenol oligomers in physiological environment [63]. Therefore, grafting modification of polyphenol is also an important method to improve the antioxidative function of bone repair materials.

(2) Dopamine-based antioxidants

**Figure 6:**
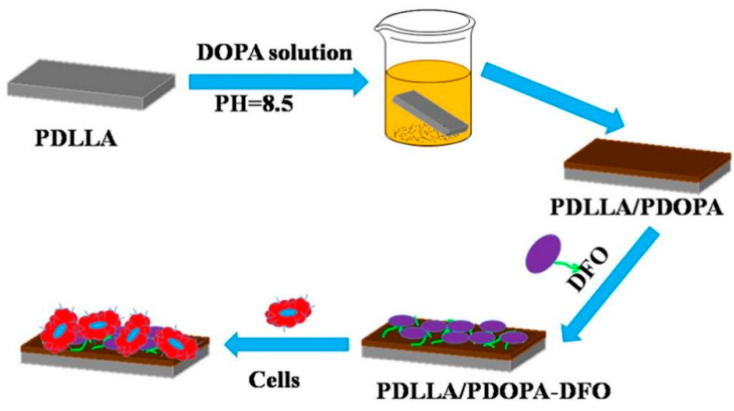
The schematic illustration of the preparation of polydopamine coating on the surface of poly(D,L-lactide) (PDLLA) membrane for loading deferoxamine (DFO) [65], Copyright, 2017, Elsevier, License Number 5627511501515.

Owning to powerful adhesion function, dopamine has been widely used in surface modification on various substrates [64,65]. As shown in Figure 6, dopamine aggregated to form a polydopamine (PDA) coating by pH regulation easily [65]. According to mussel-inspired rapid depositing process, Zhang et al. prepared the multifunctional antioxidative PDA coatings on porous and dense substrates with CuSO_4_/H_2_O_2_ as trigger [66]. In recent studies, the PDA-based antioxidative coatings were used to improve osteogenesis successfully. According to a simple in situ reduction process, Ag nanoparticle-loaded antioxidative PDA coatings were created on the surface of TiO_2_ nanotubes prepared by electrochemical anodization [67]. The incorporated PDA controlled the release pattern of Ag at low doses without long-standing cytotoxicity, and stimulated osteogenesis with desired biocompatibility [67]. Moreover, dopamine-based bioinspired coatings can be facilely modified on electrospun nanofibers of PCL [68]. Based on the process of pulsed electrochemical deposition (PED), mussel-chemistry inspired antioxidative coating was fabricated by layer-by-layer assembling in situ with polypyrrole-polydopamine nanoparticles (PPy-PDA NPs) and crystallized HA nanoparticles subsequently on the surface of porous Ti substrate from the inside to the outside [69].

(3) Chitosan-based antioxidants

Cs, a nature biomaterial similar to extracellular matrix, is a kind of partly deacetylated chitin with wide sources and well-known good biocompatibility, which is gradually applied to osteogenesis. In recent studies, Cs-based coatings, as an antioxidative substrate, exhibited great significance in bone regeneration of implants. In order to solve the diabetes-induced bone defects, Li et al. incorporated Cs (acting as an antioxidative agent) on the surface of porous Ti alloy implant via simple physical modification processes [70].

In summary, there are many kinds of organic antioxidants in bone repair biomaterials, and the properties of those antioxidants can be further improved by modification or grafting. Among them, polyphenol structure-based antioxidative biomaterials contain a large number of phenolic hydroxyl groups, showing special antioxidant activity and being critical for bone repair. However, the stability of organic antioxidants is a disadvantage, and their persistence in long-term OS environment needs to be further enhanced.

### 3.2. Inorganic Antioxidants

Compared with organic antioxidants, inorganic antioxidants are relatively stable which may contribute to antioxidation in long-term. At present, the chemical elements of inorganic antioxidants in bone repair mainly include Ce, Sr, Se, and Si, etc.

(1) Cerium-based antioxidants

**Figure 7:**
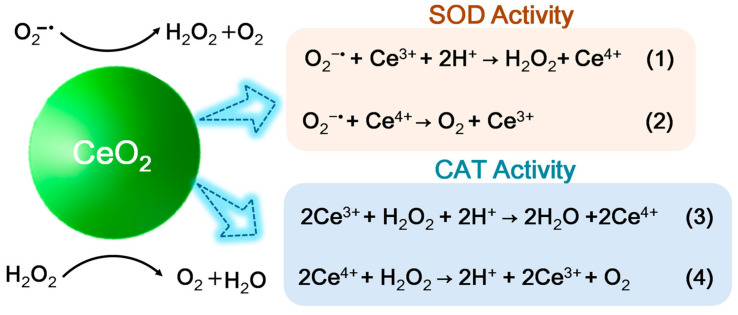
Schematic process of SOD–like and CAT–like activities of Ceria nanozyme.

Cerium is the first lanthanide metal element in the periodic table, possessing two different valent states, including Ce (III) (Xe 4f1) and Ce (IV) (the ground state of Xe). As shown in Figure 7, the fast valence switch capability of Ce^3+^/Ce^4+^oxidation states can effectively catalyze the reduction/oxidation reaction, which endows ceria nanomaterials with good mimetic activity of SOD and CAT [71,72]. Due to the enzyme-like activity, ceria in nano scale is also known as ceria nanozyme (CeO_2_NZs). Moreover, the Ce-based materials with high Ce^4+^/Ce^3+^ ratio can exhibit CAT mimetic activity, and the Ce-based materials with low Ce^4+^/Ce^3+^ ratio shows SOD mimetic activity.

BGs are functional and biocompatible silicon-based materials. In 1969, 45S5 became the first BGs prepared by L. Hench [73]. Based on various chemical compositions, many other types of BGs including 58S, 28S5, 72S, 63S, 77S, and 68S also have been produced [74], and was widely used in bone tissue engineering for enhancing bone regeneration [75,76]. Of note, the reported BGs possess favorable pro-angiogenic and pro-osteogenic activities, but a lack of antioxidative biological activity. Interestingly, Ce doping is also an important way to promote antioxidative effect of traditional bone repair materials such as BGs. Farag et al. [77] prepared BGs nanoparticles containing antioxidative Ce elements as multifunctional bone fillings, which promoted cell viability and hydroxyapatite formation. Moreover, mesoporous BGs are emerging BGs with tunable pore structure, large specific surface area (SSA) and enhanced bioactivity, showing good potential of drug delivery [78]. In order to enhance the antioxidative activity of mesoporous BGs nanoparticles (MBGN), Zheng et al. incorporated antioxidative Ce into MBGN with almost no change in pore volume and pore size [37]. Considering the unique antioxidative advantages of cerium, the preparation of Ce-based osteogenic materials have important application value.

On the other hand, owning to excellent oxidation resistance, CeO_2_ was widely applied in coating modification on Ti alloy surface for bone regeneration. Importantly, there are many suitable methods that can be used to load ceria on the surface of the substrate in a large area effectively. For instance, by magnetron sputtering of CeO_2_ target, an antioxidative cerium oxide nanoparticles (CeO_2_NPs) coating was physically bonded to Ti surface [42]. Additionally, the Ce^4+^/Ce^3+^ molar ratio and particle size of CeO_2_NPs was controlled by the deposition time of magnetron sputtering [42]. Similarly, Shao et al. [79] built cerium oxide coatings on the surface of Ti substrate with high Ce^4+^/Ce^3+^ molar ratio (namely ceria B-IV) via plasma spraying. In addition, antioxidative HA coatings incorporated by CeO_2_ were prepared onto Ti-6Al-4V substrates by plasma spraying system [43]. Furthermore, the CeO_2_-modified calcium silicate antioxidative bio-coatings (noted as CS-30Ce) on Ti-6Al-4V substrate were also successfully built by plasma-spray technique [80]. CeO_2_ incorporated calcium silicate coatings showed good ROS scavenging property and chemical stability [80]. We can see that the plasma spraying and magnetron sputtering are the main methods to modify Ti alloy with CeO_2_-based antioxidative coatings.

By sol-gel synthesis technique, CeO_2_ was incorporated in bioactive ceramics to prepare bone repair scaffold, exhibited good chemical durability [48]. In simulated body fluid (SBF), the doped antioxidative CeO_2_ in low concentration almost did not change the special property of bio-ceramics for generation and crystallization of HA layer [48]. Of note, owning to the release of Ce^2+^/Ce^3+^ in SBF, the incorporated ceria in high content increased the formation of cerium phosphate, inhibiting the growth process of HA layer formation [48]. It means the Ce incorporated bone repair scaffold is an integrated system, and the property of this scaffold can be regulated by Ce element effectively. In order to build an implantable antioxidative scaffold that possesses superior ROS scavenging properties, Kurian et al. used nanoceria (nCe) on the surface of gelatin methacryloyl (GelMA) scaffolds [81]. It can be seen that Ce-based antioxidants can be introduced into nanoparticles, surface coatings of implants and bone repair scaffolds in different ways. Ce-based antioxidants have broad application prospects and are expected to play an antioxidant role in the long term.

(2) Silicon-based antioxidants

Silicon is an important trace element in animal and human bone tissues. Many studies demonstrated the good antioxidative function of silicon-based coatings. In order to prepare a potential antioxidative biomaterial for fracture healing, surface patterned amorphous nitrogen incorporated silica (Si(ON)_x_)-based coatings were fabricated via process of plasma enhanced chemical vapor deposition (PECVD) [82]. In addition, Monte et al. prepared amorphous silica-based coating via PECVD, which effectively expressed angiogenic and antioxidant markers for faster healing and osteointegration [83]. Similarly, Monte et al. fabricated Si(ON)_x_ coatings using the PECVD method, which may be good for accelerating the vascular healing in bone defects under OS condition [84]. On the other hand, Do Monte et al. found that silicon nanocoating can further elevate angiogenesis and reduced oxidative stress, creating a favorable environment for bone repair [85]. The recent antioxidative silicon-based coatings were mainly prepared via PECVD, and exhibited great angiogenic properties and osteointegration. However, the antioxidation mechanism of those amorphous silica-based materials is not identified clearly.

(3) Selenium-based antioxidants

As an important trace element in vivo, Se also plays an important antioxidant role in human health (such as bone health) by protecting cells against OS effectively. Porous Se-treated SiO_2_ (Se@SiO_2_) nanoparticles can slowly release antioxidative Se in a controlled biosafety amount [86]. The nano porous materials are generally prepared by the doping method of antioxidative inorganic elements.

(4) Strontium-based antioxidants

Sr possesses excellent antioxidative function and high stability, which can be introduced into bioceramics via high-temperature calcination. In femoral condyle defect of ovariectomised rats, Sr-added bioglass can effectively decrease the content of MDA and inhibit the reduction in CAT, SOD and GPx activities to maintain antioxidative property against ROS-induced OS [87]. As potential antioxidative implant materials, Sr incorporated bioceramics (SiO_2_-P_2_O_5_-MgO-SrO-CaO) were synthesized via by sol-gel and calcination process, showing protection role against H_2_O_2_-induced OS in cell level [88]. Moreover, the formation rate and crystallization of surface HA layer increased with the enhancement of Sr content [88].

Although the inorganic antioxidative materials were widely used in bone repair, it is also worth noting that the biosafety of inorganic materials may be a potential problem. Some inorganic antioxidants have been reported to be cytotoxic, and the metabolism of inorganic biomaterials in vivo may not be as convenient as organic antioxidants. Moreover, the mechanism of inorganic antioxidation is not clear. In addition, Se, Sr, and other elements are trace elements with low content abundance in the body, and their large introduction in vivo may cause potential safety hazards. Therefore, there are many limitations of inorganic antioxidative materials in clinical application.

### 3.3. Organic/Inorganic Composite Antioxidant

Considering the continuous OS response in vivo, bone repair materials require long-term antioxidative effect. In order to achieve this goal, we need to rely on the efficient combination of various antioxidative components, especially the collaborative participation of organic and inorganic antioxidants. Moreover, the OS microenvironment in vivo is very complex, containing a variety of ROS and reactive nitrogen species (RNS), which needs multifunctional antioxidative materials urgently.

For meeting the requirements of bone repair under complex OS environment, Yang et al. prepared multifunctional composite CeO_2_NZs via surface modification of poly(tannic acid) (PTA) [89]. Based on the antioxidative synergy of TA and CeO_2_NZs, the PTA/CeO_2_NZs composite nanozyme featured good SOD-like activity and high free radicals scavenging activity for both ABTS^+•^ and DPPH• [89]. By against H_2_O_2_-induced oxidative damage, PTA/CeO_2_NZs effectively maintained the vitality of MC3T3-E1 cells effectively [89]. In addition, the surface PTA coating contributed to improving biocompatibility of CeO_2_NZs that enhanced cell proliferation of pre-osteoblasts and reduced the hemolysis rate of red blood cells as well. So, PTA/CeO_2_NZs with core-shell structure possess both advantages of CeO_2_NZs and TA, which shows broad application prospect in bone repair.

Under continuous OS environment, single antioxidative component is difficult to deal with the bone injury. The development and application of composite antioxidative materials needs to be further strengthened. Of note, the surface modification of organic antioxidants can enhance the antioxidative function of inorganic antioxidants and improve their biocompatibility. Compared with single antioxidants, composite antioxidants combining many organic and inorganic advantages, showing multiple antioxidative properties, which is more promising in the field of bone repair. However, the organic–inorganic composite antioxidants for bone repair are relatively few at present, needing to be further developed. Therefore, the construction of composite antioxidants is of great significance and is also an important development tendency of antioxidants in the future.

## 4. Antioxidative Properties of Antioxidative Bone Repair Materials

The introduction of antioxidants with radical scavenging effect is significant to enhance the antioxidative function of bone repair materials.

(1) Radical Scavenging Activity

**Figure 8:**
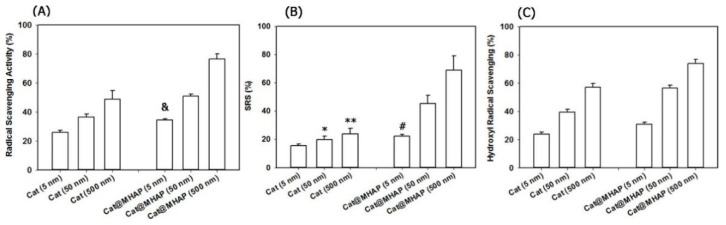
Free radical scavenging rates of catechin modified MHAP for (**A**) DPPH• radicals, (**B**) hydroxyl radicals (•OH) and (**C**) superoxide anions (O_2_^•−^) [51], Copyright, 2018, Elsevier, License Number 5627521455652. & Difference is statistically significant relative to the free catechin (5 nM) (*p* < 0.002), * Difference is not statistically significant relative to the free catechin (5 nM) (*p* > 0.05), ** Difference is not statistically significant relative to free catechin (50 nM) (*p* > 0.05), # Difference is statistically significant relative to the free catechin (5 nM) (*p* < 0.05).

Radical scavenging effect is an essential function of antioxidative bone repair materials. For instance, polyphenols are natural antioxidants with strong scavenging effect on free radicals, which have been widely used in the building of antioxidative materials for bone repair. The incorporation of quercetin exhibited strong antioxidative properties with high activity of radical scavenging for DPPH•, and completely or partially counteracted the negative effect caused by OS on the viability of osteoblast or differentiation [36]. Similarly, the catechin modified MHAP showed a high radical scavenging ability for DPPH• radicals, •OH and O_2_^•−^ (Figure 8) [51]. As reported, the DPPH• scavenging effect of modified Cs with polyphenol derivatives to was mainly resulted from the introduction of phenolic hydroxyl group and double bond [61]. Moreover, in stable composite Coll scaffolds, the Res-based macromolecular antioxidant inhibited ROS-induced cell damage of chondrocytes and bone marrow mesenchymal stem cells (BMSCs), showing good scavenging effect for DPPH• [62].

(2) Antioxidant Enzyme Mimetic Activity

OS affects the activity of intracellular antioxidant enzymes, and should not to be neglected in bone repair. Interestingly, mimic antioxidant enzymes can scavenge ROS in the outside of cells to reduce the burden of osteoblasts. For instance, CeO_2_ is a representative antioxidant that can simulate the antioxidative function of SOD and CAT, exhibiting the similar activity of antioxidant enzymes. As reported, the ceria B-IV coating showed good reduction in overproduced ROS in macrophages, and inhibited the activation of Nfkb [79]. In fact, Nfkb acted as a crucial transcription factor for macrophage regulating in immune response or inflammation [42]. Of note, the antioxidant enzyme mimetic activity of CeO_2_-based antioxidative materials was affected by the proportion of Ce^4+^/Ce^3+^. For instance, the high Ce^4+^ content of CeO_2_NPs coating showed strong CAT mimetic activity to enhance the consumption of ROS by decomposing H_2_O_2_ into H_2_O and O_2_ [42]. Therefore, it is necessary to prepare the Ce-based coatings with suitable Ce^4+^/Ce^3+^ molar ratio to enhance osteogenesis.

## 5. Biological Effects of Antioxidative Bone Repair Materials

**Figure 9:**
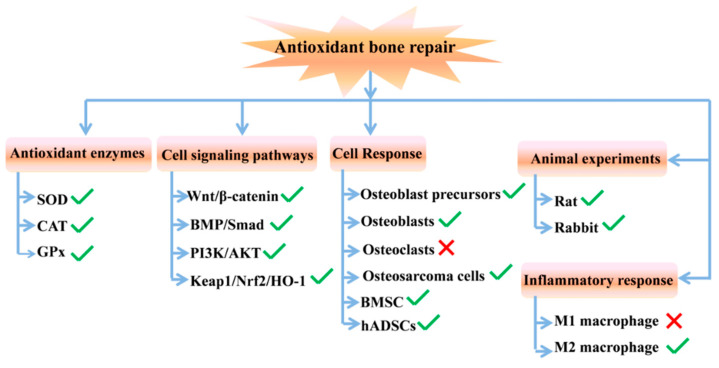
Biological effect of ABRM in different levels (the green checkmark means promoting action and the red cross means inhibiting action).

ABRM have excellent biological properties and can effectively stimulate the body responses in cellular and animal levels, as shown in Figure 9. Moreover, those antioxidative materials can regulate many signaling pathways of bone repair, and show beneficial effects on antioxidant enzymes. Interestingly, the inflammatory response can be regulated by antioxidative osteogenic materials as well.

### 5.1. Effects of Antioxidative Bone Repair Materials on Antioxidant Enzymes

In fact, the activities of antioxidant enzymes are critical to bone repair. SOD, a representative enzymatic antioxidant, is critical for collagen crosslinking, bone mineral density and even bone strength [90]. Of note, ABRM can effectively maintain or activate the activities of intracellular antioxidant enzymes. CeO_2_ incorporated HA coatings successfully maintained SOD activity, and increased cell viability in vitro with considerable decrease in oxidative injure for BMSCs by suppressed the formation level of ROS and malondiadehyde (MDA) [43]. In femoral condyle defect of ovariectomised rats, Sr-added bioglass can effectively decrease the content of MDA and inhibit the reduction in CAT, SOD and GPx activities to maintain antioxidative property against ROS-induced OS [87]. For patterned Si(ON)_x_, ROS formation was inhibited, and the enhancement of activity and expression of SOD was mainly relied on the stimulation of Si (IV) [82]. Interestingly, Si (IV) also increased the expression level of collagen matrix and bioactive HA synthesis, which was beneficial for enhancing biomineralization and osteogenesis [82]. The anti-oxidative protective vitamin E in SiO_2_ coatings can induce the activity of SOD to decreased ROS production and accumulation [44]. It is necessary to develop bone repair materials that can maintain and activate the activities of many antioxidant enzymes.

### 5.2. Effects of Antioxidative Bone Repair Materials on Cell Signaling Pathways

Of note, bone repair typically involves multiple signaling pathways. Research shows that the cellular response of antioxidative materials is obvious complex, involving the regulation of various signaling pathways. By upregulating the signaling pathway of Wnt/β-catenin, the CeO_2_ incorporated HA coatings could improve osteogenic differentiation of BMSCs against some adverse effects of H_2_O_2_ treatment [43]. In biological mechanisms, the porous nanoparticle of Se@SiO_2_ enhanced the early and late stage of osteoblastic differentiation by activating Runx2 and the signaling pathway of BMP/Smad [86]. Under diabetic conditions, antioxidant materials are critical for reducing the negative effects of mass ROS on many osteogenesis-related signaling pathways. Ma et al. prepared Cs-based nano-HA composite coating on Ti implant to enhance bone regeneration by stimulating FAK-mediated BMP-2/Smad pathway under diabetes-induced ROS overproduction [91]. The antioxidative Cs coating has considerable intra-molecular hydrogen bonding, and clearly reduced the negative effect of ROS damage mediated inhibition to PI3K/AKT pathway [70]. For diabetic culture environment, the novel antioxidative Cur/CHA scaffold may alleviate the mitochondrial dysfunction, and significantly mitigate the negative effects of cell behavior of BMSCs in migration, cell proliferation, and osteogenic differentiation [59]. By regulating the signaling pathway of Keap1/Nrf2/HO-1, the introduced Cur in Cur/CHA scaffold can effectively inhibit the diabetes caused intracellular ROS overproduction [59]. In summary, the antioxidant materials were beneficial for bone repair by regulating different osteogenic signaling pathways or molecules.

### 5.3. Effects of Antioxidative Bone Repair Materials on Cell Response

(1) Osteoblastic precursor cells

**Figure 10:**
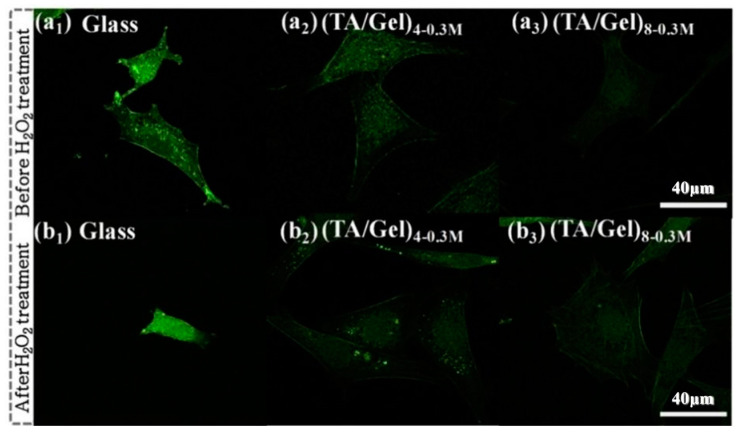
Intracellular ROS fluorescence of Intracellular ROS fluorescence on different (TA/Gel)_n_ coatings before (**a1**–**a3**) and after (**b1**–**b3**) H_2_O_2_ treatment [40], Copyright, 2019, Elsevier, License Number 5627531266472.

MC3T3-E1 cell, as a kind of mouse embryonic osteoblast precursors, has been widely used in the research of osteogenesis. MC3T3-E1 cells can specifically express osteoblast-related transcription factor and many phenotypic markers of early osteogenic differentiation (such as ALP). Research shows that the TA/Gel antioxidative multilayer coatings effectively promoted cell attachment with fastening spreading at early stage, early proliferation and osteogenesis in vitro test [40]. Moreover, TA/Gel coatings can exert good antioxidative function at cellular level by reducing intracellular ROS (Figure 10). Based on electron-transfer reaction, PDA-Ag coating effectively scavenged the excessive intracellular ROS [67]. In addition, the PDA-Ag coating showed positive osteogenic potential with increased pre-osteoblast adhesion, cell spreading and proliferation [67]. The effects of cellular regulatory was resulting from the specific surface features in nanoscale and bioactive cell-anchoring sites of PDA. Interestingly, the incorporated PDA could control the release process of Ag at low doses without long-standing cytotoxicity, and stimulated osteogenesis with desired biocompatibility [67]. In addition, the multiple antioxidative properties of PTA/CeO_2_NZs are suitable for protecting MC3T3-E1 cells under oxidative stress environment [89].

(2) Osteoblasts

In early bone healing, the Layer-by-Layer self-assembled TA/GS coating provided many attachment sites on functional nanofibers for cell recognition and adhesion with recognizable cellular pseudopodia of adhered osteoblasts [58]. For H_2_O_2_ treatment, the TA/GS multilayer showed facilitating effect of protecting osteogenic activities from ROS damage effectively and promoted cell adhesion against the down-regulated expression level of related mRNA, which was mainly attributed to the antioxidative property of abundant attached TA [58]. Similarly, as a synthetic antioxidant, catechol-conjugated Cs can protect osteoblasts against H_2_O_2_-induced oxidative damage to inhibit cell apoptosis and enhance cellular-adhesive properties by regulating the expression of cell adhesion related genes and the content anti-apoptoticrelated proteins [60]. In addition, the attached osteoblasts on HA nanofibers incorporated antioxidative Chi-C/Gel multilayer showed great cell proliferation and high cell viability with good cell spreading and clear extension feature of cytoplasmic. In vitro test, Sr introduced antioxidative BGs showed tight cell attachment with nice cell spreading morphology and stimulated cultured osteoblast to proliferate [87]. Moreover, the Sr-doped bioglass promoted osteogenic differentiation with increased formation of mineralized bone-like nodule and the bioactive layer of calcium phosphate [87]. Under diabetic environment, the deacetylated Cs coating-modified porous implant of Ti alloy showed advantageous effects for osteoblast adhesion and spreading morphology with observable filopodias and cytoplasmic extensions [70].

(3) Stem cells

Many studies have shown that antioxidative biomaterials played an important role in maintaining cell viability of BMSCs and promoting proliferation and mineralization. In nanoscale, the porous nanoparticle of Se@SiO_2_ can inhibit the ROS damage of BMSCs induced by H_2_O_2_ to protect cells withstand OS-induced apoptosis. In 2D scale, the ceria modified HA coatings can protect the early and late stage of H_2_O_2_-treated osteogenesis, increase expressions of related genes of osteogenic differentiation, and improve the activity of ALP and calcium deposition [43]. In addition, the increased surface Ce^4+^/Ce^3+^ molar ratio upregulated the expressions of osteogenic related protein and gene significantly, and even promoted the in vitro cell proliferation and osteogenic differentiation of rat BMSCs on the CeO_2_NPs samples [42]. Moreover, the bioactive PPy-PDA-HA coatings showed attractive cell affinity to BMSCs with a high level of cell adhesion, spreading and proliferation, and combined with electrical stimulation can further regulate these osteoblasts behavior [69]. Moreover, the durable PDA-HA composite coating provided favorable long-term antioxidative conditions for osteogenesis and bone regeneration against ROS caused harmful effect [69]. During the Layer-by-Layer -PED process, the integrated HA nanoparticles were subsequently in situ synthesized on antioxidative PPy-PDA assembled layer [69]. The PPy-PDA layer provided rich binding sites to combine Ca^2+^ and PO_4_^3−^, which effectively adjusted the extracellular and intracellular Ca^2+^ environment [69]. On 3D scale, the Res-based acellular scaffolds improved the cell proliferation of the cultured BMSCs. Moreover, the antioxidative Ce-based coating on the surface of GelMA scaffold provided a biocompatible environment for the growth and proliferation of BMSCs [81]. In general, antioxidative biomaterials in multiple dimensions can regulate the cell behaviors of BMSC positively.

hADSCs maintain the multi-lineage potential that can differentiate into adipogenic and osteoblasts. Owning to their advantages of wide sources and readily access, hADSCs has been widely used in bone tissue engineering. Interestingly, the antioxidant materials showed significant superiority of directionally inducing hADSCs to osteogenesis. For instance, the antioxidative catechol moiety in catechin coating was beneficial to supporting cells viability of hADSCs, enhancing in vitro mineralization, and improving osteogenic differentiation [53]. In addition, the stable catechin coatings with special surface biochemical properties can interact with serum proteins or some bioactive molecules to control cellular behavior like enhancing significantly cell viability, cellular adhesion, and spreading or even proliferative ability [53]. Similarly, the stable EGCG coating as a protective layer on the nanofibers retained effective antioxidative property to maintain cell viability and osteogenic differentiation potential of hADSCs against H_2_O_2_ mediated OS [54]. The increased hydrophilicity of synthetic polymer substrates with EGCG might be beneficial for the enhancement of cell compatibility and cell adhesion of hADSCs with great cell spreading morphology [54]. Furthermore, the bio-functional surface coating of EGCG on nanofibers positively controlled the biological functions of stem cells by inhibiting adipogenesis and enhancing osteogenic differentiation, showing great calcium deposition and mineralization [54]. For decreasing intracellular level of ROS, some chemically modified Cs porous scaffolds with high antioxidative ability were synthesized via acid-soluble/alkali-insoluble process, which were beneficial for the enhancement of the in vitro osteogenic differentiation of hADSCs [61]. Alternatively, the N-(4-hydroxyacrylamide) grafted chitosan scaffold can not only effectively increase the level of ALP expression and calcium nodules, but also improve the cell proliferation successfully, showing great biocompatibility and physicochemical activities [61].

(4) Osteosarcoma cells

**Figure 11:**
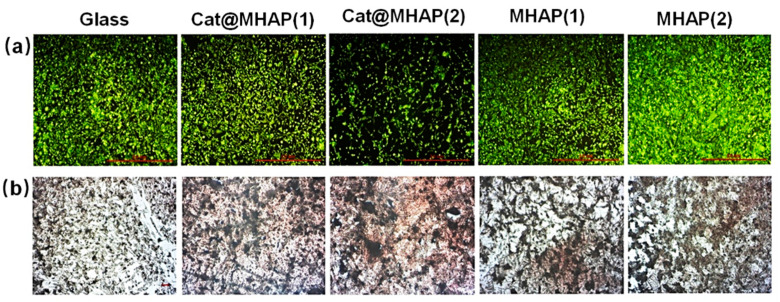
Fluorescence (**a**) and alizarin red staining (**b**) images of osteosarcoma cells on different substrate safter 14 days cultivation [36], Copyright, 2018, Elsevier, License Number 5627530717121.

Similar to osteoblasts, osteosarcoma cells also possess the ability of osteogenic differentiation, which were often selected as model cells in the field of bone repair. Some antioxidative materials can effectively regulate the behaviors of osteosarcoma cells under the environment of OS. For instance, the biocompatible sol-gel bioceramics with high content of added-Sr not only did not induce apoptosis and toxicity, but also showed great cell viability and antioxidative properties, and remarkably promoted the rate of cell proliferation in vitro for human osteosarcoma cell [88]. The biocompatible CeO_2_ incorporated CaO-P_2_O_5_-MgO-SiO_2_ bioceramics also showed great antioxidative properties, which can inhibit H_2_O_2_-induced oxidative injury and apoptotic effect to maintain high cell viability of MG-63 osteoblasts-like cell in vitro [48]. In addition, the antioxidative level of CeO_2_ modified bioceramics were increased with the enhancement of cerium dopant content [48]. However, less differentiation is one of the most significant characteristics of osteosarcoma, a malignant bone tumor with behaviors of invasion and metastasis. It is necessary to induce osteogenic differentiation of osteosarcoma cells. As shown in Figure 11, the antioxidative Cat@MHAP selectively exhibited suppressed proliferation of osteosarcoma cells and improved osteogenic differentiation in osteosarcoma cells [51]. Moreover, the OligoTyr loaded porous PLA scaffolds showed no cytotoxicity to human osteosarcoma SaOS-2 cells, and significantly exhibited a great potential enhancement of osteogenesis with efficient stimulation of ALP activity in vitro cellular systems [63]. In general, the ABRM could show promoting action for the osteogenic process of osteosarcoma cells.

(5) Osteoclasts

Osteoclasts are activated under the condition of overproduced ROS environment, which is not conducive to bone repair. However, the antioxidant materials can inhibit the activity and differentiation of osteoclasts. As reported, the EGCG-coating modified PLLA nanofiber inhibited osteoclastic differentiation by suppressing the gene expression level of osteoclast formation [54]. H_2_O_2_-treated BMSCs on the CeO_2_-sprayed HA coatings remarkably showed the inhibition of osteoclastogenesis with high mRNA expression of osteoprotegerin [43]. Moreover, the in vitro co-culture of osteoclast and osteoblast under OS microenvironment showed that quercetin loaded HA nanocrystals significantly downregulated the viability of osteoclast [35]. In addition, the results of in vitro triculture show that the incorporation of quercetin in HA stimulated osteoblast proliferation and differentiation, inhibited osteoclast viability, supported the proliferation and differentiation of endothelial cells [36].

It can be seen that the bone metabolism related cells showed various response to antioxidative materials. The stimulation of antioxidative materials can enhance the osteogenesis in vitro and/or in vivo by regulating osteoblasts behavior such as promoting cell adhesion, proliferation or osteogenic differentiation, and even inhibiting osteoclasts activity.

### 5.4. Effects of Antioxidative Bone Repair Materials on Animal Level

In vivo research, many inorganic and organic antioxidative materials showed important osteogenesis effects as well. For inorganic antioxidative materials, Se@SiO_2_ porous nanoparticle improved the healing of bone fracture in vivo experiment of SD rat [86]. On 2D scale, CeO_2_NPs coating with high proportion for Ce^4+^ improved the mineralization and osseointegration quality at the bone-implant interface [42]. The new bone tissues bonded around the CeO_2_NP-modified implant was directly and tightly without forming connective or fibrous tissue basically [42]. Under the action of patterned Si(ON)_x_ coating, the critical-sized defects model of rat calvarial was nearly totally closed with rapid generation of new bone [82]. Moreover, the regenerated bone precisely filled the gap of surrounding mature bone and patterned Si(ON)_x_-coated implant with high mineralized content in optimized biochemistry [82]. On 3D scale, in the test of in vivo osteoporotic rat model with ROS overproduction, the BG-Sr scaffolds exhibited great bone-bonding activity to increase the formation of newly formed bone matrix, during the biodegradation of the modified BGs [87]. The high content of antioxidative Sr ion released into the in vivo extracellular environment to stimulate the osteogenic processes transformed into mature bone [87].

**Figure 12:**
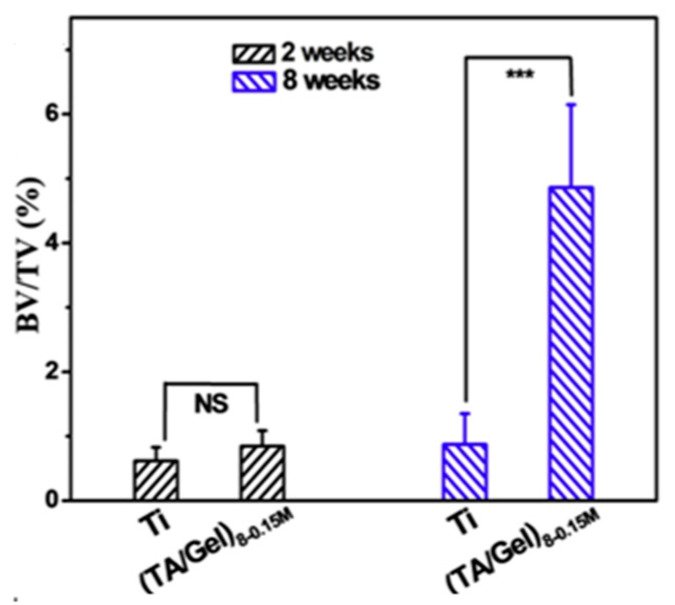
The bone volume/total volume (BV/TV) of inserted implants with and without TA/Gel antioxidative coatings modification after 2 and 8 weeks [40], Copyright, 2019, Elsevier, License Number 5627540198821. *p* < 0.01 noted as “***”, no significant noted as “NS”.

Similarly, organic antioxidative materials showed excellent effects of bone repair in vivo test as well. After hADSC transplantation, the catechin-modified nanofiber scaffolds significantly enhanced bone regeneration with high mineral density and collagen deposition in repairing the critical-sized defect of calvarial bone [53]. Moreover, EGCG-coated functional nanofibers effectively promoted the regeneration of organized and mature bone that was almost integrated continuously [54]. As shown in Figure 12, the TA/Gel antioxidative coatings could effectively improve bone regeneration and accelerate the healing of bone defect in femur of rabbits [40]. After implantation, the antioxidative surface modification on Ti with Chi-C multilayers showed great tissue compatibility [60]. With time increasing, the in vivo new bone formation at bone/implant interface and binding strength of bone-to-implant greatly increased [60]. In bone defects of diabetic sheep, it was found that the antioxidative Cs coated porous Ti implant could significantly alter the impaired osseointegration capacity of implant, showing substantial new bone regeneration in bone-implant interface, without inflammatory reaction [70]. In vivo critical-sized defect of rat calvaria model, the implanted Cur-released composite scaffolds exhibited superior osteogenic capacity that greatly increased the regeneration level of new bone and vascular with active vascular recruitment, and no extreme inflammation response was founded under type 2 diabetic condition [59].

### 5.5. Effects of Antioxidative Bone Repair Materials on Anti-Inflammation

Of note, OS is tightly related to inflammation, which may induce macrophages to polarize to M1 type to mediate inflammatory response. However, the anti-inflammatory M2 macrophage secretes osteogenic factors and inhibit inflammation to promote the process of bone repair. Interestingly, the Ce-based antioxidative coatings can regulate the cell behaviors of macrophages effectively, which exhibited potential osteogenic effects. For instance, the CeO_2_NP coating built an anti-inflammatory microenvironment by promoting the polarization of anti-inflammatory M2 macrophage phenotype. Moreover, the Ce^4+^ stimulation also abates the secretion of pro-inflammatory cytokine with improved production of healing-associated anti-inflammatory cytokine [42]. Alternatively, the CS-30Ce coatings modulated the biological polarization of macrophage by upregulating the typical surface markers expression of reparative and anti-inflammatory phenotypes (M2) considerably. Moreover, the CS-30Ce coatings significantly downregulated the mRNA expression of pro-inflammatory phenotype (M1) surface markers to reduce inflammatory responses [80]. Additionally, ceria B-IV coating effectively inhibited the polarization level of M1-type macrophages by decreasing intracellular ROS level to suppress NF-κB functions and activity. In addition, ceria B-IV coating also down-regulated the gene expression levels of M1 surface markers and downstream pro-inflammatory cytokines [79]. However, the B-IV coating was good for activating the response of M2-type macrophage with great expressing of M2 surface markers [79]. Interestingly, the mRNA expression levels of osteogenic cytokines released by macrophage were significantly up-regulated for macrophages cultured on the B-IV coatings [79]. It shows that Ce-based antioxidative coatings may regulate the behaviors of macrophages to promote osteogenesis in indirect and potential way. The biological assessments in vitro showed that Ce-MBGN reduced the expression of OS related genes significantly, inhibited inflammatory responses consequently and potentially improved osteogenic activity [37]. After implantation into the rabbit osteochondral defects in vivo, the Coll/PAA-Res composite scaffolds showed great anti-inflammatory and osteogenic activity, and finally repaired the defects perfectly with integrated neo-cartilage [62]. Therefore, it is necessary to construct ABRM with anti-inflammatory function to accelerate bone repair.

## 6. Summary and Perspectives

In the field of bone repair, bone integration is a major challenge in clinic. The overproduced ROS in bone defects greatly inhibits the process of bone regeneration and bone integration. Antioxidative function is of great significance for the development of novel osteogenic materials. Here, this review summarized the ABTS species, active units, biological responses and related biomechanisms, indicating that the antioxidative organic and inorganic antioxidants have significant and positive effects on bone repair. Of note, there are many deficiencies in present ABRM. On the one hand, the biological mechanism of antioxidant osteogenesis is really complex, involving various cells and signaling pathways, but the current research are not deep enough. On the other hand, the enduring antioxidation and suitable osteogenesis is very important for bone repair in practical application, but it is difficult to prepare high-efficient antioxidative materials with long-term and stable antioxidative effects. The future preparation of osteogenic materials should pay more attention to improving and prolonging the antioxidative activity.

## Abbreviations

Antioxidative bone repair materials (ABRM), alkaline phosphatase (ALP), Bioactive glass (BGs), bone marrow mesenchymal stem cells (BMSCs), calcium phosphates (CaP), catalase (CAT), cerium (Ce), cerium oxide nanoparticles (CeO_2_NPs), ceria nanozyme (CeO_2_NZs), chitosan (Cs), collagen/nano-hydroxyapatite (CHA), chitosan derivative conjugated with catechol group (Chi-C), Coll atelocollagen (Coll), curcumin (Cur), 2-diphenyl-1-picrylhydrazyl free radical (DPPH•), cell-extracellular matrix (ECM), epigallocatechin gallate (EGCG), gelatin (Gel), gelatin methacryloyl (GelMA), glutathione peroxidase (GPX), gentamicin sulfate (GS), hydroxyapatite (HA), human adipose-derived stem cells (hADSCs), hydrogen peroxide (H_2_O_2_), mesoporous BGs nanoparticles (MBGN), malondiadehyde (MDA), mesoporous hydroxyapatite (MHAP), nanohydroxyapatite (nHA), Singlet oxygen (^1^O_2_), superoxide anion (O_2_^−•^), osteocalcin (Ocn), hydroxyl radicals (•OH), osteopontin (Opn), oxidative stress (OS), polyacrylic acid (PAA), polycaprolactone (PCL), polydopamine (PDA), pulsed electrochemical deposition (PED), plasma enhanced chemical vapor deposition (PECVD), phenolic hydroxyl (Ph-OH), polylactic acid (PLA), poly-lactic-co-glycolic acid (PLGA), polypyrrole-polydopamine nanoparticles (PPy-PDA NPs), poly(tannic acid) (PTA), resveratrol (Res), reactive nitrogen species (RNS), peroxyl radicals (ROO•), Reactive oxygen species (ROS), simulated body fluid (SBF), selenium (Se), Se-treated SiO_2_ (Se@SiO_2_), silicon (Si), nitrogen incorporated silica (Si(ON)_x_), superoxide dismutase (SOD), strontium (Sr), Tannic acid (TA), tricalcium phosphate (TCP), titanium (Ti).
